# Historical and projected public spending on drugs for rare diseases in Canada between 2010 and 2025

**DOI:** 10.1186/s13023-022-02534-z

**Published:** 2022-10-08

**Authors:** Richard Lech, Gideon Chow, Kamalpreet Mann, Patrick Mott, Christine Malmberg, Lindy Forte

**Affiliations:** CRG-EVERSANA Canada Inc., 219 Dufferin St., Suite 210B, Toronto, ON M6K 3J1 Canada

**Keywords:** Canada, Drugs for rare diseases, Orphan drugs, Drug funding, Orphan diseases, Patient access, Public spending, Rare disease

## Abstract

**Objective:**

Rare diseases are life-threatening, debilitating, or serious chronic conditions that affect < 50/100,000 people. Canadians can only access approximately 60% of drugs for rare diseases (DRDs), which is partially related to high per-patient costs and payers’ affordability concerns. However, limiting access to DRDs can reduce survival and quality of life among patients and caregivers. Therefore, we projected Canadian non-oncology DRD spending relative to total public drug spending to provide perspective for decision makers.

**Methods:**

Candidate historical (2010–2020) and pipeline (2021–2025) Canadian-marketed non-oncology DRDs were identified using definitions from the European Medicines Agency and the US Food and Drug Administration databases. Inclusion and exclusion criteria were applied to identify eligible DRDs. Public payer claims data, prevalence rates, regulatory, and health technology assessment factors were used to project DRD spending in relation to total Canadian public drug spending.

**Results:**

We included 42 historical DRDs and 122 pipeline DRDs. Public spending on DRDs grew from $14.8 million in 2010 (11 DRDs) to $380.9 million in 2020, then a projected $527.6 million in 2021 (59 potential DRDs) and $1.6 billion in 2025 (164 potential DRDs). Projected DRD spending increased from 3.2% of $16.5 billion public drug spending in 2021 to 8.3% of $19.4 billion in 2025. These projections do not include confidential manufacturer discounts, health outcome-related offsets, or additional safety-related costs.

**Conclusions:**

Projected DRD spending shows robust growth but remains a fraction of total public drug spending. Limiting DRD access because of this growth is not aligned with Canadian patient or societal values. Given the renewed interest in a Canadian DRD framework, our results may help guide discussions that aim to balance control of public drug spending with the well-being of patients with rare diseases.

## Introduction

Health Canada defines rare diseases as life-threatening, debilitating, or serious and chronic conditions that affect a small number of individuals (< 50 cases per 100,000 population) [1]. While each rare disease has a small patient population, together rare diseases affect an estimated 2–9% of the general population [1, 2]. Rare diseases are often fatal and can have a devastating effect on life expectancy and quality of life, especially as a large proportion of rare diseases occur during childhood [3, 4]. Moreover, an Italian registry study suggested that rare diseases accounted for 4.2% of years of life lost in the general population, which was higher than the proportions for infectious diseases (1.2%) and diabetes (2.6%) [5]. Individuals with rare diseases also experience psychological and interpersonal problems due to difficulty understanding their disease, dissatisfaction with care, and limited access to treatment [6]. Therefore, rare diseases create substantial burdens on individuals and their families, and thus require an innovative healthcare approach that accounts for their unique and devastating nature.

The burden of rare diseases is difficult to overcome without approved or reimbursed treatments [1, 4] and many patients with rare diseases are left with unmet medical needs [7]. Global regulatory and reimbursement changes during the last few decades have made it increasingly viable for the pharmaceutical industry to develop innovative drugs for rare diseases (DRDs or “orphan drugs”) that may provide hope to patients who historically had few or no therapeutic options. Movement of these drugs through clinical trials, regulatory review, and health technology assessment (HTA) may help address these patients’ unmet medical needs [4].

Unlike many developed countries, including the United States (US), Canada does not have a comprehensive framework for regulatory review or public reimbursement of DRDs [1, 8–10]. This lack of a framework has contributed to Canadians only being able to access approximately 60% of globally marketed DRDs via public funding [11, 12]. Public funding of health technologies in Canada is usually reached after the completion of three steps: (1) regulatory approval through Health Canada (HC), (2) a positive HTA recommendation regarding the product’s clinical and cost effectiveness through the Canadian Agency for Drugs and Technologies in Health (CADTH) and Institut national d'excellence en santé et services sociaux (INESSS), and (3) national centralized negotiations through the pan-Canadian Pharmaceutical Alliance (pCPA) toward the achievement of risk sharing and/or confidential rebate agreements between innovators and public drug plans (federal, provincial, and territorial) [13]. Canadian patients have reported the existence of a “postal code lottery” because of province-to-province variability in reimbursement decisions, as some jurisdictions have historically delayed DRD funding or decided not to fund at all [14]. To help address this issue, the Canadian government announced plans to invest up to CAD$1 billion in a national DRD framework over 2 years starting in 2022–2023 and up to CAD$500 million per year thereafter [2, 15]. The details of the plan have not yet been publicly communicated.

Because DRDs are often significant investments on a per-patient level (sometimes exceeding CAD$0.5 million/year) [1, 2, 16, 17], there are concerns regarding potentially disproportionate resource use for DRDs in small patient populations [2]. Public payers have also expressed speculative concerns that growing DRD spending within a limited healthcare budget may affect the balance between access to effective medicine, long-term healthcare sustainability, and industry incentives [1, 2, 16]. These concerns were reinforced by the fact that a substantial proportion of patented medicines approved by HC, the US Food and Drug Administration (FDA), and the European Medicines Agency (EMA) during 2016–2018 had orphan designation from the EMA or FDA [1, 18]. In Canada, the Patented Medicines Prices Review Board (PMPRB) has noted the escalating cost of “expensive DRDs” and suggested that these products create affordability concerns [19]. However, Canadian and international studies have revealed that DRDs as a therapeutic category have a relatively low overall budgetary impact [2, 16, 20–22], accounting for < 1–9% of total pharmaceutical spending in most countries [16, 20]. One older Canadian study found that DRDs accounted for 3.3–5.6% of total pharmaceutical spending during 2007–2013 and were projected to remain at < 6% in 2018 [2]. These proportions are well within the range of other therapeutic categories and are lower than the proportions of Canadian drug spending in 2020 dedicated to oncology (15%), immunology (15%), and diabetes (9.6%) [23].

The conflicting findings and opinions regarding public spending on DRDs suggest that this issue is poorly understood and complicate efforts to develop a Canadian DRD framework [2, 15]. Up-to-date figures for Canadian DRD spending are needed to provide perspective for decision makers. Therefore, this study analyzed historical public DRD spending during 2010–2020 and projected DRD spending during 2021–2025, which were then compared to total Canadian public drug spending.

## Methods

### Classification and screening of DRDs

**Fig. 1:**
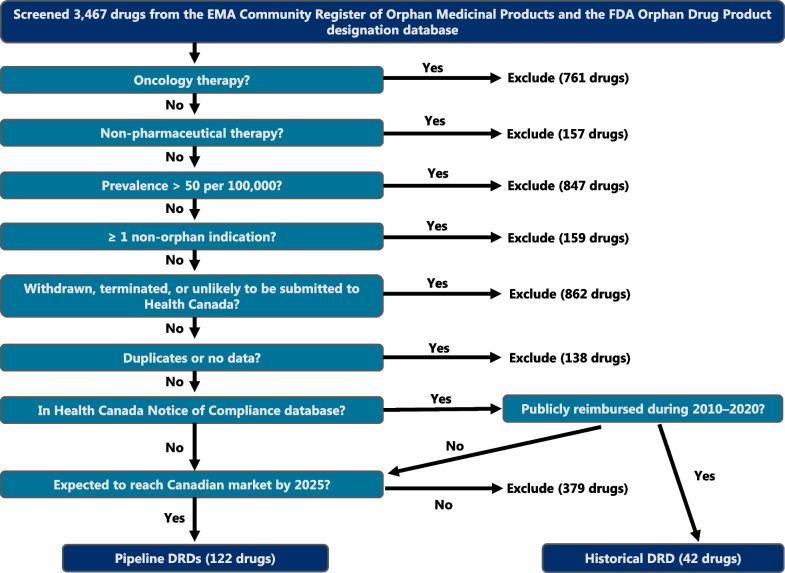
Flowchart identifying historical and pipeline drugs for rare diseases in Canada. As Canada does not have an official framework for drugs for rare diseases (DRDs), candidate DRDs were identified using the European Medicines Agency (EMA) Community Register of Orphan Medicinal Products, the US Food and Drug Administration (FDA) Orphan Drug Product designation database, and the Health Canada (HC) Notice of Compliance database. Various inclusion and exclusion criteria were applied to determine whether a DRD would reach the Canadian market by 2025. Additional assumptions used to project whether a candidate DRD would be marketed in Canada by 2025 are described in the “Budget forecasting” section. *DRDs* drugs for rare diseases, *EMA* European Medicines Agency, *FDA* US Food and Drug Administration

Because Canada does not have an official list of DRDs, candidate DRDs were identified using the EMA Community Register of Orphan Medicinal Products, the US FDA Orphan Drug Product database, and the HC Notice of Compliance database [2, 9, 24]. Candidate DRDs were defined as treatments for life-threatening diseases with a prevalence rate of < 50 cases/100,000 population [1, 2, 25], which included small molecule drugs, biologics, and gene therapies. Oncology drugs were not considered DRDs in our analysis because (1) their prices appear to not be as affected by disease rarity [26], (2) cancer treatments are managed and budgeted distinctly from other therapeutic categories in several jurisdictions, and (3) many oncology drugs are approved for numerous indications, which limits the financial impact of rare indications on overall product sales and makes it difficult to separate their use as DRDs versus standard oncology treatments [27]. Candidate DRDs were also excluded from our analysis if they were non-pharmaceutical therapies, if the prevalence of the related condition was ≥ 50 cases/100,000 population, if the drug held multiple indications that included ≥ 1 non-orphan indication, if the manufacturer withdrew or terminated their right to market authorization, and if the DRD was not expected to reach the Canadian market by 2025 (Fig. 1). Assumptions that influenced whether a DRD would potentially be eligible for public reimbursement in Canada by 2025, based on its phase of development, are described in Table 1 [28, 29]. Potentially eligible DRDs were classified as historical or pipeline DRDs.

**Table 1:** Assumptions for identifying pipeline DRDs and their first year of potential reimbursement

Current phase of development	Projected year of public reimbursement
In or completed pCPA negotiations	2021
HTA review complete	2022
FDA/EMA/HC approval, HTA review pending or in progress	2023
Completed phase 2 or 3 trial	2024
Active or recruiting phase 3 trial	2025

Assumptions are based on Lexchin et al. [29] and Government of Canada [28], plus assumptions based on professional experience

*DRDs* drugs for rare diseases, *EMA* European Medicines Agency, *FDA* US Food and Drug Administration, *HC* Health Canada, *HTA* health technology assessment, *pCPA* pan-Canadian Pharmaceutical Alliance

### Historical DRDs

Historical DRDs (2010–2020) were defined as being approved by HC for treating a rare disease (i.e., orphan designation in the EMA or FDA databases) and publicly funded at any point during 2010–2020 by at least one of the provincial drug programs (excluding Prince Edward Island, which was not included in the data set) and/or the Non-insured Health Benefits Program. Historical DRDs were assumed to be reimbursed throughout the pipeline period (2021–2025) with costs increasing in a linear manner.

### Pipeline DRDs

Pipeline DRDs were defined as DRDs that were not publicly reimbursed in Canada during 2010–2020 but were projected to potentially become eligible for reimbursement at any point between 2021 and 2025. It is important to note that the number of pipeline DRDs will likely be larger than the number of pipeline DRDs that receive a positive HTA recommendation and are actually reimbursed during this period. Examples of pipeline DRDs included (Table 1):

- therapies with orphan designation from the FDA or EMA and active phase 3 clinical trials
- therapies under regulatory review by HC, the FDA, or the EMA
- HC-approved therapies undergoing HTA review by the CADTH or INESSS
- HC-approved and HTA-recommended therapies in negotiations with the pCPA to determine confidential rebates, risk sharing factors, and Product Listing Agreements with provincial, territorial, and federal drug plans.

### Prevalence data

Published prevalence rates were used to identify “orphan” DRDs (≥ 2 to < 50 cases/100,000 population) and “ultra-orphan” DRDs (< 2 cases/100,000 population) [30, 31]. However, the rarity of rare diseases often makes it challenging to collect peer-reviewed epidemiological data. Thus, when prevalence rates could not be obtained from the literature, the search was expanded to CADTH HTA recommendations, Canadian patient group websites, the Orphanet database, and other sources.

### Data sources for public DRD spending

The PDCI Canadian Public Drug Claims Database was used to collect data regarding public spending based on list prices for historical DRDs between 2010 and 2020. For comparison, 2020 public spending based on list prices was also calculated for the top 25 drugs in Canada. Drug claims data for historical DRDs were supplemented with cost data obtained via Freedom of Information (FOI) requests to all jurisdictions covered in the PDCI data set, although responses were only received from British Columbia, Manitoba, New Brunswick, Newfoundland and Labrador, Saskatchewan, and the Non-insured Health Benefits Program. The FOI requests were performed because drug claims data may not be available for all historical DRDs, which would likely be because they were funded through special programs that were not captured within the claims database [32].

### Annual costs of historical DRDs

Total annual spending on each historical DRD (orphan or ultra-orphan) was determined using the historical claims data. Historical annual costs were separately determined for orphan DRDs (2–50 cases/100,000 population) and ultra-orphan DRDs (< 2 cases/100,000 population) because of the substantial cost difference between these two classes of DRDs [21, 33]. The number of Canadian patients who could be treated with a DRD was estimated via the prevalence rate of its associated rare disease and the population of Canada in 2020 (38,005,128) [34].

### Projecting costs of pipeline DRDs

List prices are not yet available for pipeline DRDs and it is impossible to predict which specific pipeline DRDs would become publicly reimbursed and when that would happen. Thus, annual costs were estimated for each pipeline DRD and then discounted using various assumptions that reflect attrition in the different steps between clinical trials and public reimbursement (Table 2) [11, 20, 24, 35, 36]. First, publicly available list prices and prevalence data were used to calculate average prevalence-weighted per-patient costs for historical DRDs, which were found to be CAD$103,774/year for orphan DRDs and CAD$356,279/year for ultra-orphan DRDs. The prevalence values for pipeline DRDs were then used to categorize them as orphan or ultra-orphan drugs. Next, the annual number of patients who were projected to be treated with a pipeline DRD was calculated using the disease-/indication-specific prevalence data. Finally, the annual cost of treating each pipeline DRD’s projected patient population was calculated by multiplying the number of treated patients by the DRD’s average annual prevalence-weighted per-patient cost (i.e., CAD$103,774/year for orphan DRDs and CAD$356,279/year for ultra-orphan DRDs). While this approach estimates all possible future spending, it does not reflect real-world factors, such as regulatory and HTA failure rates. Therefore, we applied discounting factors (Table 2) to each DRD’s total possible future cost based on its current phase of development (Table 1). For example, the cost of a pipeline DRD in pCPA negotiations would be discounted based on Canadian market penetration, public/private split, and a half-cycle correction in the first year (i.e., assumptions related to FDA/EMA/HC approval and HTA recommendation do not apply because these milestones have been passed). In contrast, the cost of a pipeline DRD with an active or completed phase 3 trial would be discounted based on all assumptions, as it has yet to receive FDA, EMA, or HC approval. This approach is generally consistent with a similar European analysis conducted by Schey et al., albeit with some differences in methodology and parameter definitions [20]. For example, Schey et al. included assumptions regarding loss of marketing exclusivity/patent protection and the introduction of new orphan drugs for indications that are already covered by existing orphan drugs, which resulted in a flattening of the growth curve [20]. We were unable to account for these factors in our projections and thus our results do not account for any flattening of the DRD growth curve.

**Table 2:** Assumptions for predicting the future costs of pipeline DRDs

Assumption	Parameters
Only a subset of DRDs will be approved by the FDA or EMA after completing a phase 3 trial [35]	66.3% of DRDs with a completed phase 3 trial are approved, 33.7% are not approved
HC often delays/does not grant approval to DRDs that are approved by the FDA or EMA [11, 36]	HC approval rates were estimated as 16% after 1 year, 30% after 2 years, 40% after 3 years, 50% after 4 years, and 60% after 5 years
Some DRDs are not recommended for reimbursement despite HC approval [24]	69.15% of DRDs would receive a positive HTA recommendation, 30.85% would receive a negative recommendation
Canadian market penetration of reimbursed DRDs will increase over time [20]	DRDs capture 10% of the market in the first year of reimbursement, 12% in year 2, 15% in year 3, 20% in year 4, and 25% in year 5
Public and private payers pay for DRDs [assumption]	60% of costs borne by public payers, 40% borne by private payers
A half-cycle correction is applied during the launch year [assumption]	DRDs will capture 50% of their eligible market during the launch year

Assumptions for predicting future costs of a pipeline DRD were applied based on the DRD’s status in Table 1 (e.g., the FDA/EMA/HC and HTA assumptions would not be applied to a product in pCPA negotiations, as this product has already received regulatory approval and a positive HTA recommendation)

*DRDs* drugs for rare diseases, *EMA* European Medicines Agency, *FDA* US Food and Drug Administration, *HC* Health Canada, *HTA* health technology assessment, *pCPA* pan-Canadian Pharmaceutical Alliance

### Budget forecasting

Annual public spending on historical DRDs between 2010 and 2020 was calculated as the sum of all claims-based public spending on historical DRDs each year. Projected annual public spending on DRDs between 2021 and 2025 was calculated by (1) linearly extrapolating the annual costs for historical DRDs and (2) adding the projected annual costs for pipeline DRDs from 2021 to 2025. To simplify the analyses, we did not consider savings due to cost offsets (e.g., reduced costs of managing comorbidities and adverse events) [21], or the potential for additional costs (e.g., to manage safety issues). Therefore, the base case cost projections are based on historical data, public list prices, and no cost offsets or incidental costs.

### Scenario analyses

Because of inherent uncertainty in some of our assumptions, six scenarios were considered to determine their effects on projected public DRD spending during 2021–2025. First, only historical DRD costs were projected, which ignored the contribution of pipeline DRDs during 2021–2025. Second, confidential negotiations between the manufacturer and payers typically reduce the actual costs (vs. list prices), so a hypothetical 35% rebate was applied to the projected DRD costs. Third, HTA recommendations play a major role in public reimbursement of DRDs, so the rate of positive recommendations was raised from 69.15 to 90%. Fourth, the average annual per-patient ultra-orphan DRD cost was increased to the simple average of CAD$435,000/year (population-weighted average: CAD$356,000/year). Fifth, the average annual per-patient orphan DRD cost was increased to the simple average of CAD$175,000/year (population-weighted average: CAD$104,000/year). Sixth, annual prevalence-weighted per-patient costs were re-calculated for historical DRDs that first achieved public reimbursement during the most recent 3-year period (2018–2020, orphan DRDs: CAD$180,244/year, ultra-orphan DRDs: CAD$457,133/year) and the pipeline analysis was updated to account for the possibility that newer DRDs may have higher costs than older DRDs.

### Canadian public drug spending

Total Canadian public drug spending data from the Canadian Institute for Health Information (CIHI) were available for 2014–2020 (CAD$15.82 billion in 2020) [37]. Future public spending was estimated in a linear manner each year up to 2025 using the annual data from 2014 to 2020.

## Results

### Numbers of historical and pipeline DRDs

A total of 3467 candidate DRDs were screened (Fig. 1). The most common exclusions were based on withdrawal of orphan drug status or product termination, non-orphan indications being held by the candidate drug (e.g., malaria), and oncology drugs. A total of 42 historical DRDs with HC approval and public claims data were included in the analysis. The screening process also identified 510 DRDs that might be eligible for future reimbursement in Canada. Applying the assumptions regarding the first year of potential reimbursement (Table 1) predicted that 122 pipeline DRDs would potentially be eligible for public reimbursement by 2025.

Approximately 4 historical DRDs became eligible for public funding each year during 2010–2020 and approximately 24 pipeline DRDs were predicted to potentially be eligible for public reimbursement each year during 2021–2025. This difference appears substantial. However, it is important to note that the projected cost for a given year did not capture full reimbursement of approximately 24 pipeline DRDs, as we cannot predict future reimbursement on a case-by-case basis (see “Methods” section, “Projecting costs of pipeline DRDs” section). While 24 pipeline DRDs might be potentially eligible for reimbursement in a given year, a much smaller number would actually be reimbursed based on the assumptions in Table 2, especially in later years where the pipeline includes DRDs that have not received HC, FDA, or EMA approval.

### Public spending on DRDs

**Fig. 2:**
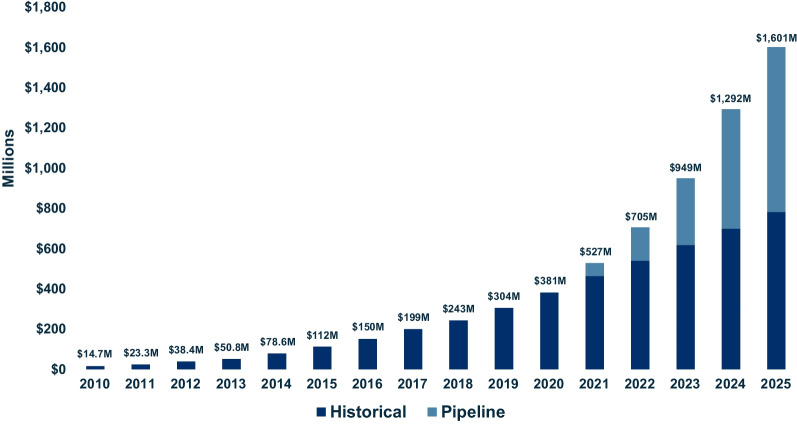
Annual spending on historical and pipeline drugs for rare diseases between 2010 and 2025. Public spending on Health Canada-approved historical drugs for rare diseases (DRDs) was evaluated from 2010 (11 DRDs) to 2020 (42 DRDs). Linear extrapolation was used to project spending on historical DRDs during 2021–2025, and projected spending on pipeline DRDs was added to produce the total annual spending on historical and pipeline DRDs during 2021–2025. All amounts are shown in millions of Canadian dollars. Assumptions that guided our projections for pipeline DRDs are listed in Tables 1 and 2

Public spending on historical DRDs grew from CAD$14.8 million in 2010 (11 DRDs) to CAD$380.9 million in 2020 (41 DRDs). Projected public spending on historical and pipeline DRDs grew from CAD$527.6 million in 2021 (59 potential DRDs) to CAD$1.6 billion in 2025 (164 potential DRDs) (Fig. 2).

**Fig. 3:**
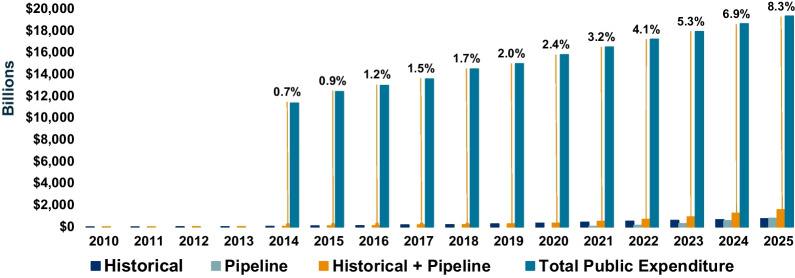
Spending on drugs for rare diseases as a proportion of total public drug spending. Historical data were available for total public drug spending (billions of Canadian dollars) from 2014 to 2020, and linear extrapolation was used to project values out to 2025. Spending on historical, pipeline, and historical plus pipeline drugs for rare diseases (DRDs) is shown, with the proportion of spending on historical plus pipeline DRDs shown in relation to total public drug spending for each year

To place this growth in DRD spending into perspective, public DRD spending was evaluated as a proportion of total public drug spending. Historical data showed that total public drug spending increased from CAD$11.4 billion in 2014 to CAD$15.82 billion in 2020, with linear extrapolation used to project out to 2025. As proportions of total public drug spending, DRD costs increased from 0.7% of CAD$11.4 billion in 2014 to 3.2% of CAD$16.5 billion in 2021 and 8.3% of CAD$19.4 billion in 2025 (Fig. 3). The increase in public DRD spending reflects the increasing number of treatments that are becoming available for patients with rare diseases, although the overall cost remains a fraction of public drug spending. It is also important to note that these projections are based on list prices for historical DRDs and do not account for confidential rebates from the manufacturers, cost offsets from improved patient health outcomes, or incidental costs (e.g., because of safety issues).

### Scenario analyses

The effects of changing some of the base case assumptions were evaluated in six scenario analyses, which showed that public DRD spending as a proportion of total public drug spending ranged from 4.0 to 10.3%. Considering only historical DRD costs (without any pipeline DRDs) projected an increase in DRD spending to $782.8 million in 2025 (4.0% of total public drug spending). Applying a hypothetical 35% discount to list prices projected an increase in DRD spending to CAD$1.04 billion in 2025 (5.4% of total public drug expenditures). Assuming a very high rate of positive HTA recommendations (90% vs. 69.15%) projected an increase in DRD spending to CAD$1.75 billion in 2025 (9.0% of total public drug spending). Increasing the average annual per-patient ultra-orphan DRD cost to CAD$435,000/year projected an increase in DRD spending to $1.65 billion in 2025 (8.5% of total public drug spending). Increasing the average annual per-patient orphan DRD cost to CAD$175,000/year projected an increase in DRD spending to CAD$2.00 billion in 2025 (10.3% of total public drug spending). Using annual prevalence-weighted per-patient costs for historical DRDs that achieved reimbursement in the most recent 3-year period (2018–2020, orphan DRDs: CAD$180,244/year, ultra-orphan DRDs: CAD$457,133/year) projected an increase in DRD spending to CAD$2.09 billion in 2025 (10.8% of total public drug spending).

### DRDs are rare among drugs with the highest public spending in Canada

**Fig. 4:**
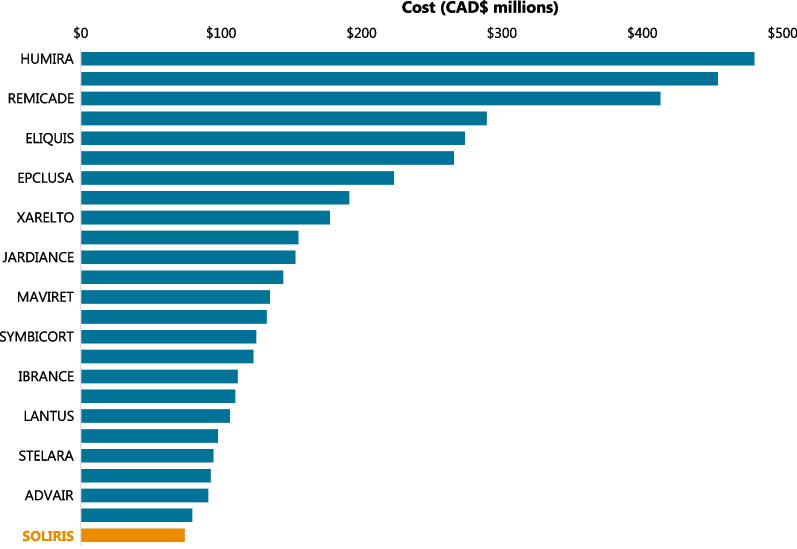
Public spending on the top 25 drugs in Canada during 2020. Historical data were collected for the top 25 drugs in Canada during 2020 (costs in millions of Canadian dollars) to examine the number of drugs for rare diseases (DRDs) in this list. Despite the high per-patient costs of DRDs, only 1 of the top 25 drugs was a DRD (SOLIRIS®, eculizumab) and its annual cost was dwarfed by the costs of drugs for more common conditions

Historical data were also used to identify the top 25 drugs in 2020 according to Canadian public drug spending (Fig. 4). The only DRD in this list was SOLIRIS® (eculizumab, CAD$74 million) and its cost was dwarfed by other commonly used drugs, such as HUMIRA® (adalimumab, CAD$480 million), EYLEA® (aflibercept, CAD$454 million), and REMICADE® (infliximab, CAD$413 million).

## Discussion

The high per-patient costs of DRDs have led to concerns regarding whether DRD funding disproportionately directs healthcare resources to small patient populations and potentially affects the balance between access to effective medicines, long-term healthcare sustainability, and industry incentives [1, 2, 16]. Our study evaluated historical and projected public spending on non-oncology DRDs to evaluate whether these concerns are based on the available evidence. The results projected an increase in public DRD spending from CAD$14.7 million in 2010 (11 DRDs) to CAD$1.6 billion in 2025 (164 potential DRDs). It is also noteworthy that only one DRD (SOLIRIS®, eculizumab) was present in the 2020 list of the top 25 drugs according to public spending. A similar analysis from 2018 shows that spending on this DRD has increased (2018: CAD$54 million, 2020: CAD$74 million), although it has slipped from #18 in 2018 to #25 in 2020 among the top drugs according to public spending [38]. Public DRD spending is also dwarfed by total public health spending (e.g., on drugs, hospitals, physicians, and medical services), which was CAD$267 billion in 2019 [39]. Furthermore, almost 40% of total public drug spending in Canada is for approximately 2% of beneficiaries, with little or no public outcry, and approximately 60% of these beneficiaries use drug therapies that cost CAD$10,000 or more per year [37].

The increase in the number of potential DRDs during our study period reflects recent and rapid innovation leading to new treatments for historically under-served populations of Canadian patients with previously untreatable rare diseases. While the overall increase in spending may seem large, the increase is much more modest when considered in relation to total public drug spending (from 0.7% of CAD$11.4 billion in 2014 to 8.3% of CAD$19.4 billion in 2025). In a similar European analysis, Schey et al. projected that orphan drug spending as a proportion of total drug spending would increase from approximately 0.5% in 2004 to 4.6% in 2014 (a 9.2-fold increase) [20]. A naïve comparison shows numerically consistent 10-year increases in our results (9.9-fold for 2014–2024 or 9.2-fold for 2015–2025), albeit in different periods that may reflect the relative delay of DRD marketing in Canada versus Europe. Interestingly, Schey et al. included various assumptions that resulted in a flattening of the growth curve for orphan drug spending (e.g., introduction of generics/biosimilars and competition within indications) [20]. It is conceivable that a similar flattening dynamic could play out in Canada, although we currently lack data to model the related parameters.

To place public spending on DRDs into perspective, it is useful to consider spending on other therapeutic categories. An IQVIA report estimated that the proportions of Canadian drug spending in 2020 according to therapeutic categories were approximately 15% for oncology, 15% for immunology, 9.6% for diabetes, 5.9% for neurology, 5.9% for mental health, and 5% for cardiovascular [23]. The Canadian proportions in 1995 from the same report were approximately 3.7% for oncology, < 1% for immunology, 3% for diabetes, 5.9% for neurology, 6.7% for mental health, and 20% for cardiovascular [23]. These temporal changes reflect the drug lifecycle of innovation, initial robust investments in patent-protected brands, a shift to off-patent and generic versions, and then new innovations (often in different therapeutic categories) [23, 40]. Periods of innovation do not appear to destabilize drug budgets as the proportion of drug spending versus healthcare spending has remained relatively steady during 1995–2020 [23], despite increasing reliance on pharmaceuticals drugs during this period. Therefore, periods of innovation and robust investments in specific therapeutic categories (e.g., DRDs) are likely to be fluid and temporally limited processes [23], which are unlikely to be unsustainable or to destabilize public drug spending or the Canadian healthcare system.

Our study is timely because the Canadian government recently renewed discussions regarding a national strategy to improve market and patient access to DRDs [1, 14, 15], which has been a point of contention in Canada. For example, HC initially rejected the idea of a DRD policy in 1997, announced plans to develop a regulatory framework in 2012, subsequently removed all references to these plans in 2017, and then started public consultations on a framework in 2021 [14, 41]. Increased access to DRDs has been speculatively linked to unsustainable growth in public drug spending, which has led to an unpredictable and restrictive HTA environment that would strongly discourage pharmaceutical companies from bringing new DRDs to Canada based on recommended price reductions of 32–99% [42]. However, our findings suggest that concerns regarding unsustainable growth in public DRD spending are not justified, as the increase in public DRD spending still only accounts for a fraction of overall public drug spending and is dwarfed by total public health spending. This information can inform evidence-based discussions regarding a Canadian DRD framework that can improve patient access without straining the Canadian healthcare budget.

Our analyses provide evidence that DRDs will not drive unsustainable growth in Canadian public drug spending, as our screening was rigorous and our projections based on available evidence. The rigor of our screening is demonstrated by the fact that all our historical DRDs were included in a list of 93 “expensive drugs for rare diseases” (including oncology drugs, which we excluded) that was published by the PMPRB in 2020 [19]. Furthermore, our projections were largely based on list prices, which are typically discounted via confidential manufacturer rebates, and did not consider cost offsets from improved patient outcomes with DRDs. Moreover, our projections likely overestimated the contribution of pipeline DRDs to annual public drug spending, as spending on each pipeline DRD was forecast independently of other drugs that might be used for the same patient populations, which could have led to double counting. Finally, our projections did not account for the introduction of generic or biosimilar DRDs over time. Therefore, our results may be overestimates that do not yet reflect the growth curve flattening that was predicted in a similar European study [20].

The present study has several limitations that should be acknowledged. First, our pipeline projections are based on several assumptions and changes in these parameters over time might increase or decrease the projected costs of pipeline DRDs. Second, our projections are largely based on claims data for historical DRDs and it is possible that complete data might not be available for some historical DRDs [32]. We attempted to mitigate these issues using FOI requests, although the resulting data were incomplete and may not have captured all public spending. Third, we assumed that public spending on historical DRDs would increase annually in a linear manner, although there is year-to-year variability in public spending on historical DRDs due to changing drug prices (e.g., because of inflation, competition, and patent expiration) [20, 21, 43]. Furthermore, public spending on a historical DRD may increase due to greater disease awareness, improved diagnostic methods, and growth in the eligible population if the treatment is not curative. Fourth, we simplified the analyses by not considering other healthcare spending that could be avoided or incurred with DRD treatment, such as resources to manage clinical events and co-morbidities [21], or the potential for additional costs to manage safety issues. Fifth, our analyses did not incorporate measures of therapeutic effect (e.g., survival or quality-adjusted life-years) that would be needed to examine the cost-effectiveness of DRDs (e.g., based on incremental cost effectiveness ratio).

## Conclusions

Our findings indicate that DRDs will account for only a modest proportion of total public drug spending in Canada up to 2025. These projections are likely overestimates because they do not consider confidential manufacturer discounts, cost offsets from improved patient health outcomes, or the possibility of generic/biosimilar DRDs entering the Canadian market. Therefore, concerns regarding unsustainable growth of public spending on DRDs appear to be unwarranted, as this growth reflects the normal market dynamic of innovation and investment in historically under-served patient populations. Limiting publicly funded access to DRDs based on these concerns is also not aligned with patient values and has created an unfair “postal code lottery” that determines which patients can access life-saving or life-changing treatments [14]. Given the renewed interest in a Canadian DRD framework, our results may help guide discussions that aim to balance control of public drug spending with the well-being of patients with rare diseases.

## Data Availability

The data that support the findings of this study are available from PDCI Market Access but restrictions apply to the availability of these data, which were used under license for the current study, and so are not publicly available. Data are however available from the authors upon reasonable request and with permission of PDCI Market Access.

## Abbreviations

CADTH: Canadian Agency for Drugs and Technologies in Health
DRDs: Drugs for rare diseases
EMA: European Medicines Agency
FDA: Food and Drug Administration
FOI: Freedom of information
HC: Health Canada
HTA: Health technology assessment
INESSS: Institut national d'excellence en santé et services sociaux
pCPA: Pan-Canadian Pharmaceutical Alliance
PMPRB: Patented Medicines Prices Review Board
US: United States
